# Food insecurity and coping strategies in international migrants in transit through Mexico

**DOI:** 10.1016/j.jmh.2022.100099

**Published:** 2022-04-04

**Authors:** Caroline Irene Deschak, Cesar Infante, Verónica Mundo-Rosas, Alondra Coral Aragón-Gama, Manuela Orjuela-Grimm

**Affiliations:** a Mexican School of Public Health, Colonia Santa Maria Ahuacatitlan, National Institute of Public Health, Av. Universidad 655, Cuernavaca, Morelos CP 62100, México; b Center for Health Systems Research, Colonia Santa Maria Ahuacatitlan, National Institute of Public Health, Av. Universidad 655, Cuernavaca, Morelos CP 62100, México; c Center for Evaluation and Survey Research, Colonia Santa Maria Ahuacatitlan, National Institute of Public Health, Av. Universidad 655, Cuernavaca, Morelos CP 62100, México; d Center for Nutrition and Health Research, Colonia Santa Maria Ahuacatitlan, National Institute of Public Health, Av. Universidad 655, Cuernavaca, Morelos CP 62100, México; e Department of Epidemiology and Pediatrics, Columbia University Medical Center, 722 west 168th Street, Rm 730, New York, NY 10032, United States

**Keywords:** Migration, Food security, Food access, Coping, Mexico, Transit

## Abstract

**Background:**

Although migrants in transit through Mexico have been known to consume insufficient food, data measuring food insecurity (FI) in this vulnerable population is scarce. This study assesses FI in migrants transiting Mexico by examining economic, physical and social food access.

**Methods:**

Using a mixed-methods design and a convenience sample of migrants residing in a temporary shelter in Saltillo, Coahuila in northern Mexico, we applied 1) the Food Insecurity Experience Scale (adapted), and 2) a semi-structured interview exploring FI coping strategies.

**Results:**

Of the 54 participants, 74.1% reported moderate or severe FI and 24.1% reported severe FI, especially reduced food quantity. Interviewees reported poverty, cargo train travel, and crime as FI contributing factors. All interviewees used FI coping strategies, principally altering food intake and using social resources. Many strategies reported implied negative consequences for both nutrition and general health.

**Conclusions:**

This study provides a comprehensive assessment of FI in a mobile population using a standard international measure. Results confirm widespread and severe FI, highlighting a potential humanitarian crisis requiring both national and international attention. Further research is critical to inform strategies for guaranteeing the right to food access for migrants in transit through third (non-destination) countries.

## Highlights

• Globally, data measuring food insecurity among migrants in transit is scarce.
• 74.1% of migrants in this study reported moderate or severe FI, especially reduced food quantity.
• FI coping strategies used by migrants implied negative nutrition and general health implications.
• The widespread and severe FI in migrant highlights a potential humanitarian crisis in the region.
• Further research is critical to inform strategies for guaranteeing the right to food access for migrants.

## Introduction

Access to food is part of the universal human right to an adequate standard of living (Food and Agriculture Organization of the United Nations (FAO) 1996) and key target of United Nations (UN) Sustainable Development Goal 2 (United Nations Development Program (UNPD) 2020). Most nations around the world, including Mexico, guarantee the right to food access within their borders, especially for priority groups and those in vulnerable conditions such as mobile or displaced groups. International migrants, who constitute 3.5% of the world's population (International Organization for Migration (IOM) of the United Nations 2019), are increasingly vulnerable as restrictive immigration policies and scarce social capital limit their access to food and other basic necessities during the migratory process [(Subsecretary of Population Migration and Religious Affairs 2011, Food and Agriculture Organization of the United Nations (FAO) 2019, Red de Documentacion de las Organizaciones Defensoras de Migrantes (REDODEM) 2019)]. Despite legally-binding commitments within Mexico's Constitution, Migration Law, and other legislation guaranteeing the universal right to food access (Federal Government of Mexico 1917, Subsecretary of Population Migration and Religious Affairs 2011), migrants in Mexico have reported insufficient food access (Aragón Gama et al., 2020); however, knowledge of the prevalence and consequences of this insufficiency is limited.

International migrants in Mexico face structural conditions that perpetuate social inequity and marginalization and exacerbate their vulnerability (Stoesslé et al., 2015, Doctors Without Borders of Mexico and Central America 2019, Leyva-Flores et al., 2019, Infante, Aggleton and Pridmore, 2009). Annual flows of irregular migrants transiting Mexico increased more than twofold from 2010 (128,400 migrants) to 2017 (296,800 migrants) with 90% originating in Central America. In this same period, migration policies in Mexico have become increasingly militarized, prioritizing national security over migrant rights (Villafuerte, 2017). Violence towards migrants in Mexico has been widely documented and has manifested in multiple ways, including psychological, physical and sexual, with repercussions for health and wellbeing (Leyva-Flores et al., 2019, Infante et al., 2020). Food security and violence are closely linked, with organized crime networks in Central America and Mexico directly limiting economic and physical access to food through extortion and theft [(National Human Rights Commission of Mexico (CNDH) 2018, International Organization for Migration (IOM) 2015)]. Notably, crime can also serve as a coping strategy for obtaining food or basic necessities (International Organization for Migration (IOM) 2015).

In addition to crime, migrants and asylum seekers in Central America and Mexico confront other significant risks posed by socioeconomic inequities, natural disasters, food shortages, and barriers to healthcare and justice system access (UN 2022, ACAPS 2020). Within this context, civil society largely bears the burden of providing humanitarian and other assistance to mobile groups, but its capacity for risk mitigation without State support is limited. Despite these compounded challenges, the UN does not currently classify the migration phenomenon in Mexico as a humanitarian crisis (ACAPS 2020, United Nations 2017).

The historic level of need brought on by these regional circumstances has led Mexico to sign multiple key international agreements which aim to guarantee the rights of mobile groups. Among these is the 2018 Global Pact for Safe, Orderly and Regular Migration, which recognize the rights of migrants within the countries through which they transit or where they settle. However, state actions are insufficient to adequately implement these international accords, resulting in the neglect of the critical needs of mobile populations (Archibugi and Held, 2012, Maldonado Valera and Marinho y C. Robles, 2020).

Humanitarian assistance for migrants transiting Mexico is primarily provided by civil society organizations that operate shelters (*casas del migrante*). Migrant shelters, though each is unique, generally offer a variety free goods and services including food, shelter (day or overnight), clothing, basic health care, and legal support for immigration or administrative procedures [(Red de Documentacion de las Organizaciones Defensoras de Migrantes (REDODEM) 2019, Cervantes and Muñoz, 2016, National Institute for Migration 2018)]. Evidence indicates that most healthcare services sought by migrants in transit are provided in shelters (Instituto Nacional de Salud Pública de México 2016). It is unknown how many migrants use the network of approximately 96 shelters located along the main migratory routes through Mexico, but the socio-demographic profile of their users is similar to that reported for detained migrants returned to their countries of origin by Mexican authorities (Cervantes and Muñoz, 2016, Li Ng, 2020), supporting the idea that the profile of migrants who use shelters is reflective of a large proportion of all migrants in overland transit.

Despite the presence of shelters and limited governmental assistance, there is mounting evidence of inadequate food access for international migrants transiting Mexico, including acute hunger and coping strategies normally reserved for emergencies (Aragón Gama et al., 2020, Stoesslé et al., 2015, Sheridan, 2019). A 2016 survey of international migrants in shelters across Mexico revealed that 65.6% consumed insufficient food during their transit, and some relied on strategies such as foraging and begging in order to eat (National Human Rights Commission of Mexico (CNDH) 2018). A recent study reported decreased food intake in 74% of migrants during transit through Mexico, including 33% who reported one or more consecutive days without eating (Aragón Gama et al., 2020). These studies have explored key aspects of severe food security, but without a systematic exploration of the range of experiences necessary to inform effective approaches to address it.

Food security is a key component of wellbeing and is understood as “physical, social and economic access to sufficient safe and nutritious food that meets dietary needs and food preferences for an active and healthy life” (Food and Agriculture Organization of the United Nations (FAO) 1996). It therefore encompasses both quantity and quantity of food consumed, as well as mental processes such as worrying about obtaining food which are critical to the comprehensive understanding of food access. Food security is multidimensional, and considers food availability, accessibility (economic, physical and social), utilization and stability over time. However, the food access dimension is of particular relevance to populations in conditions of vulnerability due to specific barriers limiting access for certain individuals or groups even when sufficient food is available at the national or regional level (Food and Agriculture Organization of the United Nations (FAO) 1996). The inverse of food security is food insecurity (FI), understood as the failure to fully achieve food security through socially acceptable means, possibly including physical hunger symptoms. The prevalence of moderate or severe FI is one of two indicators used globally to track hunger and indicates a failure to fully achieve the right to sufficient and adequate food access [(Food and Agriculture Organization of the United Nations (FAO) 1996, Federation of American Societies for Experimental Biology of the American Institute of Nutrition 1990)].

Although standardized quantitative tools are widely applied in the general population in order to measure the economic access subdimension of food security, data on physical and social food access are limited (Deschak et al., 2021). All three subdimensions of food access can be assessed through the analysis of food insecurity coping strategies, understood as individual actions implemented with the goal to acquire and maintain food in acceptable ways (Maxwell and Caldwell, 2008). Coping strategies differ widely depending on available resources and cultural considerations, and therefore reveal economic, physical and social barriers to food access in a given context including resorting to socially unacceptable means to attain food (Lazarus and Folkman, 1984, Global Partners, 2019). Among overland migrants in Mexico, these may include known risk factors such as geographical isolation, discrimination, and organized crime [(Red de Documentacion de las Organizaciones Defensoras de Migrantes (REDODEM) 2019, Doctors Without Borders of Mexico and Central America 2019)]. Coping strategies, while not inherently harmful, may negatively affect health and wellbeing (e.g., when they exacerbate poor dietary patterns or expose the individual to illness or violence).

The objective of this study was to analyze food insecurity in international migrants transiting Mexico through a comprehensive analysis of the magnitude and severity of inadequate food access and associated coping strategies, in order to characterize factors affecting the rights to health and wellbeing during migration.

## Material and methods

Data collection took place in the *Casa del Migrante de Saltillo* (CDMS), which since 2002 has provided migrants transiting Mexico with goods and services including food, clothing, overnight shelter, basic health care, hygiene, legal and administrative support, local job placement, and recreational activities. The CDMS serves migrants from highly diverse cultural backgrounds, though most are from the northern triangle of Central America. Located in Saltillo, Coahuila, the CDMS is situated near the tracks of the North-South freight train line which traces one of the most highly-frequented migrant routes in Mexico (the Gulf route), at approximately 85 kilometers from Monterrey, Nuevo Leon, and 300 kilometers from the US-Mexico border.

This study analyzes food security through the economic, physical and social subdimensions of food access using a mixed methods design for development (Greene, Caracelli and Graham, 1989), in which results of the quantitative phase guided implementation of the qualitative phase. Two instruments were applied sequentially. The first was a survey comprising sociodemographic information and the Food Insecurity Experience Scale (FIES, individual-level Spanish-language module). The FIES is a standardized tool to measure food security by economic access using eight variables, each represented in one question about food insecurity experiences: for example, eating only a few kinds of foods, or feeling hungry but not being able to eat (Food and Agriculture Organization of the United Nations (FAO) 2020). The FIES is the monitoring instrument for UN Sustainable Development Goal indicator 2.1.2 (United Nations Development Program (UNPD) 2020), and measures economic food access by associating self-reported experiences with a score reflecting FI severity: null, mild, moderate or severe [(Consejo Nacional de Evaluacion de la Polica de Desarrollo Social (CONEVAL) of Mexico 2010, Maxwell and Caldwell, 2008)]. The FIES survey module was applied with an adaption to the reference period, where the original period of either 30 or 365 days was modified to reflect the amount of time each participant had spent transiting Mexico before the survey.

The second study instrument was a semi-structured interview designed to explore economic, physical and social food access through seven guiding questions exploring coping strategies used to prevent or mitigate food insecurity while in Mexico, and their health-related consequences (Maxwell and Caldwell, 2008, Ballard, Kepple and Cafiero, 2013, Dil Farzana et al., 2017, Kempson et al., 2003, Anater, Nonnemaker and McWilliams, 2012). Probes on the specific coping strategies used were derived from the Coping Strategies Index [3024]. Survey participants with preliminary FIES results indicating severe food insecurity during transit were later invited to participate in the interview by convenience sampling, according to their continued presence in the CDMS. The interview allowed a deeper exploration of their experiences and of how their actions to combat food shortages compared to existing evidence on negative coping strategies. All coping strategies were organized into four categories and six subcategories: 1) social resource strategies, either a] formal (organizations offering food assistance) or b] informal (individuals offering non-systematic food assistance); 2) food consumption strategies altering either a] quality or b] quantity of food intake; 3) financial strategies which a] augment income or b] limit non-food expenditures or; 4) other. Negative coping strategies were considered to be those which implied harm to health or wellbeing including livelihoods; the usage and frequency of these strategies generally increase alongside FI severity. Available evidence (Lazarus and Folkman, 1984, Ballard, Kepple and Cafiero, 2013, Dil Farzana et al., 2017, Kempson et al., 2003, Anater, Nonnemaker and McWilliams, 2012) guided the themes explored in the interviews, which included, during transit through Mexico: difficulties obtaining food and perceived causes, primary and secondary strategies for obtaining food, pre-existing health conditions, perceived health consequences of limited food access and coping strategies, and risks related to use of coping strategies. In the case of participants traveling with one or more minors, we also explored strategies used to guarantee food for the child or adolescent and their potential impact on the adult's own food access.

### Participants and data collection

The first author (CD) collected data over three weeks in March 2020, but fieldwork ended prematurely due to COVID-19. Eligible participants were international migrants ≥18 years old who used the CDMS during the study period and reported the intent to transit Mexico to reach a third country. Those who had spent ≤5 days in Mexico were ineligible. Users of the CDMS were invited to participate during group gatherings including meals, workshops, and entrance interviews. Consenting individuals were invited to complete a written self-administered survey with facilitation as needed by CD. Survey responses were immediately reviewed and participants indicating moderate to severe FI or negative coping strategies were considered eligible for the semi-structured interview (30-60 minutes). Of eligible interview participants, females or those traveling with minors were prioritized for selection in order to promote representation by key priority groups. All survey participants who were invited to participate in an interview accepted.

The final objective of ≥60 surveys and ≥15 interviews reflected the minimum of data expected to ensure sufficient information to highlight trends and reveal new concepts in non-representative study circumstances. Sampling was by convenience based on study scope, expected feasibility based on previous study experiences [(Red de Documentacion de las Organizaciones Defensoras de Migrantes (REDODEM) 2019, Aragón Gama et al., 2020)], and historical migratory flow to the CDMS during the study period (on average, 388 overnight guests during the month of March between 2016-2020) (Manzo, 2021). Participation was voluntary and anonymous, and required informed verbal consent. Final analysis included 54 surveys and 10 interviews.

This study was approved by the Ethics Committee and Institutional Review Board of the National Institute of Public Health of Mexico. A code was assigned to each questionnaire and audio recording file to maintain anonymity and confidentiality of informants. No incentive, economic or otherwise, was provided to participants.

### Data processing and analysis

FIES responses were scored from 0-8 by raw number of affirmative responses according to FAO recommendations for non-representative study circumstances, where 0=null FI, 1-4=mild FI, 5-7=moderate FI and 8=severe FI (ACAPS 2020). The aggregate category of global food insecurity included respondents with moderate or severe FI (raw score 5-8). Due to the limited sample, Rasch scores could not be calculated. Scores and sociodemographic characteristics were entered into an Excel spreadsheet. Descriptive analysis was performed using Stata 14.0 (STATA 2019). Of the 59 surveys collected, five were excluded due to incomplete or illegible responses, leaving 54 for final analysis.

Semi-structured interviews were conducted, transcribed and analyzed in Spanish. The objective of the semi-structured interviews was to further understand FI experiences and coping strategies used by international migrants in the Mexican context. Several processes derived from grounded theory were used for analysis, including list coding, open coding and category construction (Strauss et al., 1994). Primary codes were constructed *a priori* to explore FI coping strategy type, frequency and determining factors. New codes were added throughout data collection to reflect emerging themes and relevant categories referenced by interviewees. To enhance reliability, members of the research team regularly discussed the coding scheme, category and theme development, and data interpretation (Lincoln and Guba, 1985).

The FIES results provided a standardized quantitative measurement of FI through economic access, while the interview results allowed a qualitative exploration of economic, physical and social access. Combined results contributed data on multiple facets of food access allowing a comprehensive analysis of the FI experience during transit through Mexico.

## Results

### Descriptive characteristics

Table 1 shows complete descriptive characteristics of the international migrants in transit who participated in the survey. Survey participants were mainly comprised of men (83.3%) from Honduras (68.5%) with an average age of 31.5 years, and had had spent a median of 60 days in transit through Mexico at time of survey. Approximately 40.1% had been in the country for less than 31 days.

**Table 1:** Demographic and migratory characteristics of Central American migrants in transit surveyed (N=54).

**Participant characteristics**	**Subgroupings**	**n**	**%**
Age (years)^a^	18-24	19	35.2
	25-39	22	40.1
	≥40	13	24.1
Sex	Male	45	83.3
	Female	9	16.7
Country of origin	Honduras	37	68.5
	Guatemala	5	9.3
	El Salvador	5	9.3
	Nicaragua	3	5.6
Destination country	United States of America	32	59.3
	Mexico	13	24.1
	Other	3	5.6
Education	≤Primary^b^	34	63.0
	Secondary^b^ or technical degree	18	33.3
	Superior (university or higher)	2	3.7
Time in Mexico (months)^c^	≤1	22	40.1
	1-3	10	18.5
	3-12	13	24.1
	>12	9	16.7
Travel unit	Traveling alone	25	46.3
	Traveling with others	29	53.7
Accompaniment by minors(age <18 years)	Not accompanied by minors	36	66.7
	Accompanied by 1-3 minors	16	29.6
	Accompanied by ≥ 4 minors	2	3.7
Health problem during transit?^d^	Yes^e^	31	57.4
	No	22	40.1
Health care receivedduring transit?^f^	Yes	29	94.0
	No	2	6.0
If health care wasreceived, where?^g^	Migrant shelter	21	66.0
	Public hospital/health center	8	26.0
	Private hospital/health center	1	3.0
	Multiple types of services used	6	19.0

a Mean (SD)=31.5 ± 10.7, [(ACAPS 2020),60]

b Primary education was considered through 6th grade. Secondary school was considered as 7th-12th grade.

c Median=2.03, [15, 6935 days]

d One participant (2.5%) did not respond.

e Some responses to this open-ended question were subject to interpretation. Approximately, of those participants who reported a health condition during transit, the majority were acute respiratory infections, followed by injuries, and general pain and malaise. A smaller number reported gastrointestinal distress, non-transmissible chronic diseases and possible vector-borne illnesses. Several female participants were pregnant and/or gave birth during transit (n=2).

f Responses expressed as a function of participants reporting ≥1 health problem during transit (n=31). Multiple responses were possible.

g Responses were expressed as a function of participants reporting ≥1 health problem during transit and having received health care during transit (n=29)

### Food insecurity by standard quantitative measures

Table 2 shows that 74.1% of migrants experienced moderate or severe food security as measured by the FIES, indicating an inadequate quality or quantity of food intake during transit through Mexico. The responses of nearly one quarter (24.1%) of migrants surveyed reflected the most severe grade of food insecurity, which includes 24 or more consecutive hours without eating due to lack of resources.

**Table 2:** Food insecurity among international migrants in transit surveyed, as measured by FIES raw score (N=54).

**Severity of food insecurity**	**n**	**%**
Null	3	5.6
Mild	11	20.4
Moderate	27	50.0
Severe	13	24.1
Global*	**40**	**74.1**

*Global food insecurity is defined as the sum of participants with moderate or severe food insecurity.

The most frequent FI experiences involved insufficient food quantity: feeling hungry but not eating (79.6%), eating less than perceived as necessary (79.6%) or skipping a meal (77.8%), all due to lack of economic resources (data not shown).

### Food insecurity determinants

Among the survey participants who were also interviewed, three contextual factors were highlighted as principal determinants of FI and negative coping strategies during transit. First, poverty limited economic food access; interviewees had few economic resources before migrating, and this condition was aggravated during transit. Secondly, use of the cargo train as transportation limited physical and social food access, particularly when transiting remote areas. Finally, crime affected economic and social food access, with nearly all interviewees experiencing physical violence and/or armed robbery during transit. Crime decreased economic and material resources, even provoking participants to not carry food to avoid targeting, and affected coping strategies by generating distrust towards social resources.

### Food insecurity coping strategies

**Fig. 1:**
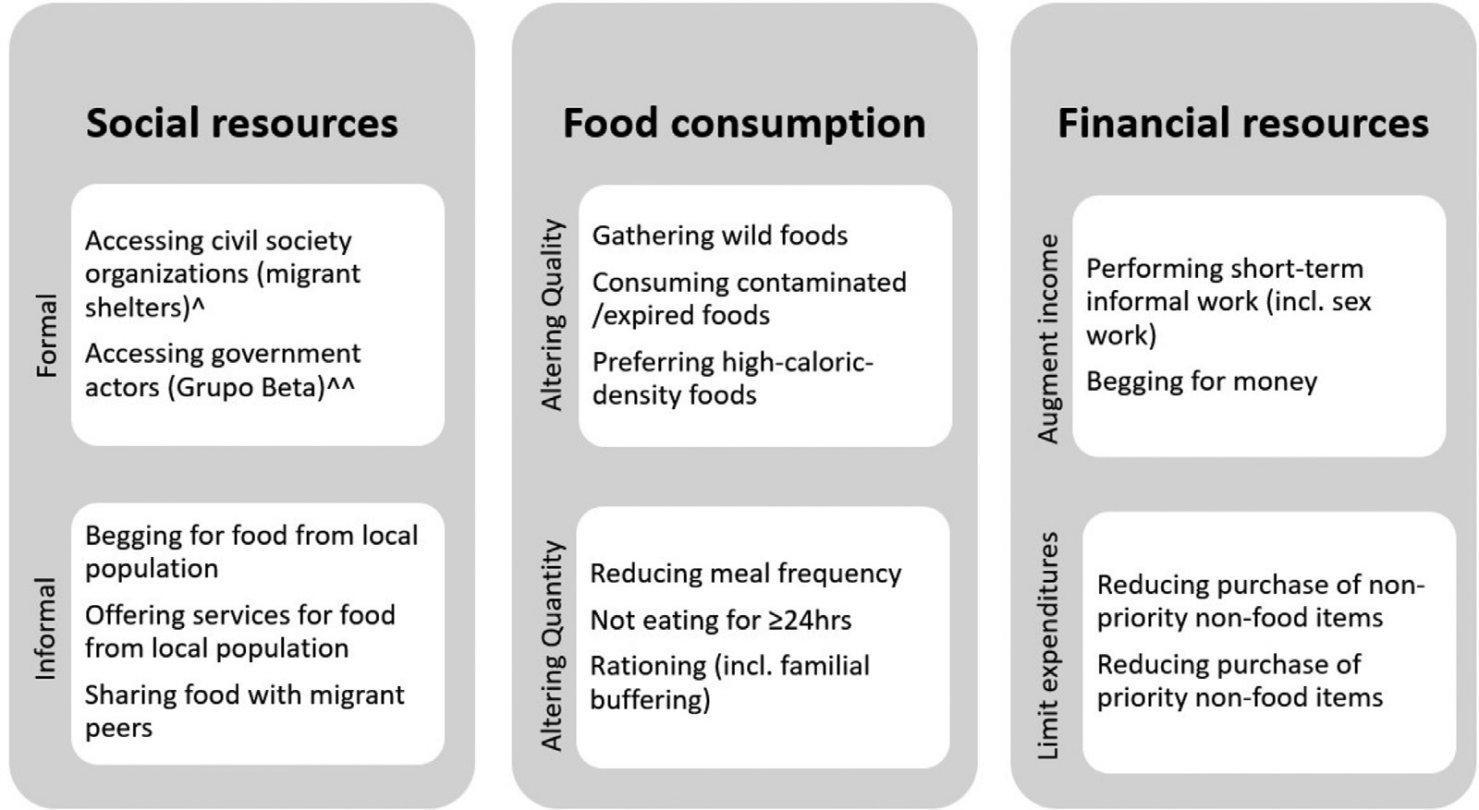
Food insecurity coping strategies reported by international migrants in transit interviewed, by category and subcategory (n=10).

^Migrant shelters in Mexico are spaces run by civil society organizations which provide a range of basic humanitarian assistance to migrants in Mexico (generally to migrants in transit). ^^Grupo Beta is a dependency of the Mexican government's Instituto Nacional de Migración (National Institute of Migration) under the Ministry of Governance, the latter of which has the mandate of protecting and guaranteeing the human rights of migrants. Grupo Beta supports by provide information on migratory processes and assisted return, and performing rescue and first aid for migrants independent of migratory status in Mexico.”

All interviewees reported using FI coping strategies, which differed geographically. The south of Mexico reportedly offered more opportunities for gathering wild food and obtaining informal social assistance, whereas the north offered better labor opportunities. Fig. 1 lists the coping strategies described by interviewees.

The following subsections explore the strategies in Fig. 1, by category.

### Coping strategies–Social resources

Interviewees obtained food through social resources, principally civil society organizations and individuals, sometimes judging the safety of a potential source by religious beliefs.

“I have always trusted in God, and let it be through him that I choose the right people to help me through this long - and honestly – terrible, savage journey. […] I try to look for help from places like the houses of Christians, a church, a cathedral, and I approach those people because I can trust them”

– 29-year-old Honduran man, over 12 months in transit

Religion implied trustworthiness, and often formed a selection criterion for choosing safe sources of assistance within an environment where violence, theft and kidnapping were common threats. However, despite the perception of Mexico as the most dangerous country in the region, it was recognized as providing a unique social resource through its migrant shelters.

“In Mexico you have to find migrant shelters. We've stayed in [at least 7] where they gave us food, drink, lodging […] Now I'm here [in the CDMS] and they treat us well, I've eaten beans, rice, bread, vegetables, chicken, three meals a day. We have enough to drink, a place to sleep, I feel like a king [*laugh*]. I can go one, two, three days without eating [on the train and walking] and I don't get sick as long as afterwards I can eat in a shelter [*laugh*]. Once I ate four plates of food when I arrived, because I had just gone three or four days on the road without eating.”

– 29-year-old Honduran man, 3-12 months in transit, travelling with two minors (ages 2 and 8)

Migrant shelters represented an indispensable source of affordable (free) and reliable food, in terms of both quantity and quality. They provided hot meals prepared in kitchens, and usually offered more variety and more unprocessed choices than food items obtainable through personal resources or piecemeal donations from local individuals. Shelters represented the only reliable point of both food assistance and physical safety along the migrant route, and migrants used few other formal social resources due either to the complete absence of these, or distrust in using them.

Nearly all interviewees also reported relying heavily on locals for food: an informal social resource. This took the form of begging for food or offering services in exchange for food.

“The people of Mexico take care of our hunger […] if one of us [migrants] doesn't have resources like money, food, we can go to a house and ask for a taco and people will give you food. […] Sometimes you can tell people don't like you very much, there's some racism … but there are always others, just civilians on the street, who help you because they know on the road we suffer and sometimes we don't eat. […] I'm thankful to all the people who help us on the road, they're the angels that feed us.”

-54-year-old Honduran man, less than one month in transit

This strategy was not always successful and some interviewees reported receiving violent or discriminatory reactions to requesting food assistance from individuals. Nonetheless, in general interviewees expressed gratitude for the generosity of local populations in providing food when asked, particularly in more rural localities in the southern states of Mexico.

### Coping strategies–Food intake

All interviewees reported strategies, which diminished dietary quality by limiting dietary diversity or nutrient balance, especially outside the shelter context. Even more frequently, migrants significantly reduced food intake through rationing, skipping mealtimes, and by not eating for days at a time.

“I had no money, and I sometimes I asked people and they wouldn't give me food. I've gone more than a day, almost two days [without eating]. You just have to wait and wait until you find a little food to eat, or someone gives you some. I had to do it many times, just put up with the hunger until I could get to a migrant shelter.”

-21-year-old Honduran woman, 1-3 months in transit, pregnant and travelling with one minor (age 2)

Frequent reports of a complete lack of food intake for ≥24 consecutive hours, which confirm and expand upon that documented quantitatively by the FIES (see section 3.2), directly reflect a lack of adequate food access and the dependence on limited external resources to satisfy basic needs. Interviewees provided further detail to these experiences by describing how they or their peers chose not to eat as a strategy to avoid crime or preserve resources.

“In the places we walk, even when I'm hungry and thirsty I won't go with just anyone who says, “Come here, we'll give you food”. […] That's how people take advantage of our hunger and thirst on the road, that's why we don't trust anyone anymore. First, they give you food, then something to drink, then they say, ‘This is a kidnapping. Cooperate or die.’ […] On top of that, if you bring water [and food] on the train and don't share people may beat you up just to get your water bottle.”

-51-year-old Honduran man (1), 1-3 months in transit

“Some people do carry money, and they still don't buy food. They'll tell you they don't have anything but when robbers come, they pull out money [to pay them]. How can you have money and still let yourself go hungry? Me, if I have money, I'm won't go hungry. You can't eat money … it comes and goes. My health comes first.”

-54-year-old Honduran man, less than one month in transit

Without sufficient internal resources, and with safe external resources practically confined to small-scale charitable assistance, migrants transiting Mexico described an environment where achieving adequate food access was nearly impossible.

In the case of adults accompanying minors, all used “buffering”, where adults prioritized ensuring the food needs of children over their own needs.

“The [two-year-old] girl, I prefer she eat instead of me. As long as there is food, she eats something. Sure, on the road we suffer hunger, the girl too. Once none of us ate for three or four days. But [as a guardian] you don't eat so that the little ones can eat instead. That's the answer.”

– 29-year-old Honduran man, 3-12 months in transit, travelling with two minors (ages 2 and 8)

The use of this strategy demonstrated an overarching priority towards the wellbeing of children and adolescents, at the expense of accompanying adults.

### Coping strategies–Financial resources

Financial strategies were infrequent, purportedly due to lack of economic liquidity and income, despite the desire to work and some involvement in temporary small-income-generating activities in Mexico. Nonetheless, some interviewees reported manipulating scarce financial resources to mitigate hunger, such as by reducing non-food expenses in priority areas such as transportation and healthcare.

“I have a daily medication for hypertension. I get it from the migrant shelters [or] I ask people on the street, but it's really difficult, sometimes I can't get both medicine and food. If I were to just get the medicine and not have anything in my stomach, well … food is the priority.”

-29-year-old Honduran man, over one year in transit

In this case, the strategy of reducing medical expenditures to buy food illustrates a lack of access to the rights to both food access and health services during transit through Mexico, and implies greater risks for those individuals with chronic health conditions. Nonetheless, food was consistently prioritized, even at the expense of other purchases critical to health and wellbeing.

### Other coping strategies

Several strategies reported did not correspond to any category in Fig. 1, and related to different ways of securing food or resources for food. For example, many interviewees reported stealing fruits, vegetables and cereals (corn) to satisfy hunger.

“Yes, we stole [crops] shamelessly, but we only do it if we really have to, just a little to help us get by. Almost all the train tracks have corn fields nearby, so when we're hungry we grab three [ears] here, continue then grab three more, and that's how we eat.”

-21-year-old Honduran man, 3-12 months in transit

Multiple interviewees expressed strong disapproval of theft as against their moral standards, and this minor theft from fields and marketplaces was not perceived as an extralegal activity but a survival strategy. However, it could provoke legal and social consequences such as arrest, sanction, or rejection by the local community.

Other strategies included dressing in tattered clothing to preserve resources by avoiding being targeted by thieves and, for those who began their journey alone, to seek accompaniment by migrant peers in an effort to improve chances of procuring food through resource and labor sharing. The latter did not necessarily imply participation in “migrant caravans”.

### Health consequences of negative coping strategies

Nearly all coping strategies reported implied negative consequences for health and wellbeing, spanning categories including and beyond nutrition, such as: food safety, physical integrity, financial stability, legal standing and social wellness. For example, the consumption of contaminated food when it was the only thing available affected food safety.

“Many of us leave our country clean and healthy, but on the road many get sick […], especially stomach problems. On the train many don't take care of themselves because of hunger and exhaustion. Some bring food and leave some of it abandoned on the train; if you find it you eat it. Sure, I've gotten sick … normal things, like stomach problems. The common illnesses we always confront in life.”

-51-year-old Honduran man (2), over one year in transit

This strategy was widely understood to cause gastrointestinal illnesses, such that these were normalized as part of migration. Furthermore, physical and social consequences resulted from high-risk tasks to obtain food or food money, including sex work.

“There are good things and bad things about selling your body. You make easy money, but instead for 200 pesos you can get AIDS, or an infection. [But] if I didn't give something in return no one would give me anything. I had nothing to eat, so even if I didn't like it, I had to do it to get a bite of food. [And] to save a child from doing the same. If they want to rape me they can, but I would never let my ten-year-old [sister] do the same.”

-22-year-old Nicaraguan man, over one year in transit, traveling with four minors (unknown ages)

Sex work was not directly reported by migrants traveling without minors, but was reported by one individual traveling with minors, potentially revealing consequences that are of greater concern for mixed-generation travel units. Finally, many other strategies reported including begging and minor theft, may aggravate discrimination against migrants, further reducing access to social assistance and increasing exposure to crime.

## Discussion

Our results highlight the severity of food insecurity among migrants transiting Mexico using an international standard instrument, including frequent use of negative coping strategies.

Seventy-four percent of participants (N=54) experienced moderate or severe FI during migration; well above even that of Mexican population sectors with similar characteristics of vulnerability such as poverty (Mundo Rosas et al., 2019, Hill et al., 2011). The magnitude of FI reported is also similar to estimates of up to 82% previously described in US migrant agricultural workers (Smith, Rabbitt and Coleman- Jensen, 2017), reinforcing evidence of international displacement as a determinant of FI. By using the FIES to measure food access, our results were consistent with prior evidence of insufficient food intake in migrants documented using other methodologies [(Red de Documentacion de las Organizaciones Defensoras de Migrantes (REDODEM) 2019, Stoesslé et al., 2015)]. This supports the validity of our results while highlighting the specific relevance of ensuring food quantity as a focus for migrants in transit.

Our study reinforces poverty as a determining factor of FI, as previously documented worldwide (Sadiddin, Cattaneo and Cirillo, 2018, Tsegaye et al., 2018). However, our findings also highlight the role of factors more specific to the Mexican context, such as the well-documented conditions of violence and the use of the cargo train as a mode of transportation [(International Organization for Migration (IOM) of the United Nations 2019, Stoesslé et al., 2015)], and the negative impacts of these on food access. Our findings affirmed previous testimonies from migrants in transit through Mexico showing widespread theft of economic resources (National Human Rights Commission of Mexico (CNDH) 2018), and contributed evidence of theft of basic goods including of food itself.

A key FI coping strategy for migrants during transit is accessing social resources: notably, migrant shelters and local individuals with whom migrants have no previous relationship. These networks are distinct from those commonly used by settled (non-mobile) populations, which often rely on family, friends and neighbors, and may also include multisector social programs which support livelihoods and which are largely absent in the transit migration context (Anderson et al., 2018, Asesefa Kisi et al., 2018). Mexico's unique network of migrant shelters, as well as other non-governmental support organizations, is key to mitigating FI during transit (Cervantes and Muñoz, 2016, Li Ng, 2020). The shelters provide not only food, but a “safe space” and comprehensive assistance including healthcare and labor opportunities, thereby minimizing the need for negative coping strategies and guaranteeing a basic human right.

Similar to settled populations (Dil Farzana et al., 2017, Asesefa Kisi et al., 2018, Kruger, Schönfeldt and Owen, 2008), food consumption coping strategies were among those most frequently employed by migrants. However, migrants primarily report alterations in quantity rather than quality of food consumed, often fasting for multiple days. In Latin America, violence has been reported as the principal driver of food insecurity (Sadiddin, Cattaneo and Cirillo, 2018); fasting with the objective to minimize potential interactions with criminals is a notable strategy reflecting the direct influence of regional violence on individual food security. In addition, adult migrants accompanying minors reported, “maternal buffering”: a common strategy in settled populations, which exacerbates FI among mothers (Castillo Ramírez, 2018). However, our findings in migrants revealed the use of this strategy by maternal and non-maternal figures, highlighting the non-traditional family structures present during migration, and additional barriers to food security for any adult traveling with minors.

Finally, migrants reported minimal use of financial strategies due to limitations in livelihood attainment, material assets, baseline solvency and access to financial services. Whereas settled populations frequently cope with FI by liquidating material assets and accessing loans or credits (Maxwell and Caldwell, 2008, Asesefa Kisi et al., 2018, Kruger, Schönfeldt and Owen, 2008), migrants in international mobility generally lack these financial resources. The limited capacity to manipulate financial resources likely contributes to greater reliance by migrants on other types of coping strategies like manipulating food consumption and tapping social resources.

Consequences of negative coping strategies go beyond poor nutrition, harming multiple facets of wellbeing. Reported strategies such as sex work and theft may lead to direct health consequences including disease and abuse, but also to the exacerbation of already-widespread stigma and discrimination against migrants in Mexico (Leyva-Flores et al., 2019). Given the demonstrated centrality of social resources to the wellbeing of migrants in transit (Stoesslé et al., 2015, Cervantes and Muñoz, 2016), as reinforced by our results, factors which damage the public perception of migrants may be particularly harmful as they limit social resources, which mitigate food insecurity.

The condition of migrants in transit through Mexico has been described anecdotally and in the media as a humanitarian crisis [(International Organization for Migration (IOM) of the United Nations 2019, Doctors Without Borders of Mexico and Central America 2019)]. One global classification system defines a food-related crisis as a general population showing ≥20% moderate or severe household FI as measured by the FIES, or in which emergency coping strategies are employed (Global Partners, 2019). In our migrant sample, FI exceeds both these criteria, providing evidence towards the classification of a food crisis in the key international migration corridor through Mexico and highlighting the need for humanitarian action. Undoubtedly, the magnitude and severity of FI documented in this study reveals the broader work required of government agencies to effectively protect the human rights of migrants. In order to meet the current basic needs of mobile groups, such work must extend far beyond the current humanitarian assistance provided by civil society organizations and international cooperation (Archibugi and Held, 2012, Cruz, 2019, United Nations Economic Commission for Latin America and the Caribbean (ECLAC) 2020).

The COVID-19 pandemic declared in March 2020 has drastically impacted the Americas, creating immobility, isolation and uncertainty for international migrants. In 2020, the economy of the Latin American region contracted by 7.7% (World Food Program (WFP) 2020), and throughout 2021 FI is predicted to increase by 269%, with international migrants disproportionately affected [(International Organization for Migration of the United Nations IOM and World Food Program WFP, 2020),58]. Although migration slowed during 2020, it increased dramatically in early 2021 and is expected to continue rising, with FI as a major push factor aggravated by the consequences of the pandemic and climate change [58]. These sobering estimates highlight the urgent need to monitor FI among the growing numbers of migrants in conditions of extreme vulnerability, in order to ensure equity in the right to food access. The ever-expanding number of international migrants worldwide is generally not accounted for within population-level food security estimates, however, evidence from our study and previous literature [(Red de Documentacion de las Organizaciones Defensoras de Migrantes (REDODEM) 2019, Stoesslé et al., 2015, Villafuerte, 2017, Deschak et al., 2021)] indicates that they represent a population of extreme vulnerability and an upper limit of these measures. It is critical that they be estimated in order to accurately measure global goals to achieve “Zero Hunger” and identify those population groups being left behind (United Nations Development Program (UNPD) 2020).

Key limitations of this study included its small sample size due to premature termination of data collection, and the inability to pilot the instruments, both of which restricted possibilities to test the internal validity of the instruments using the Rasch model. Our adaptation of the FIES included a variable reference period, which limited statistical validity but facilitated its capacity to capture relevant food insecurity experiences during the unique process of migratory transit. Our study achieved its goal of providing a comprehensive analysis of FI in migrants transiting Mexico, however, further study is needed to standardize a definition of “migrant in transit” and validate an instrument for use within this highly vulnerable population group. Larger studies, supplemented by large-scale data on dietary quality and humanitarian needs, will provide critical inputs to national and international food security indicators necessary for appropriate humanitarian response and policy-making.

## Conclusions

This study represents one of the first comprehensive measurements of food insecurity in a mobile population including the use of adapted international standard tools. It expands upon previous estimates of hunger in migrants in transit through Mexico, using quantitative and qualitative data to provide an integrative analysis of economic, physical and social food access considering regional context and potential health consequences. Our results show that migrants transiting Mexico are not guaranteed food access as stipulated by national law, and support classifying their situation as a food crisis necessitating multisectoral responses to alleviate immediate humanitarian needs and the enactment of mechanisms to operationalize the existing legislation. Though the existence of a food crisis is critical data to consider in the classification of a general humanitarian crisis, the design and scope of our study does not systematically explore other key elements needed to determine the existence of a humanitarian crisis as related to this unique population group. Further research, particularly the validation of practical and comprehensive food security measurement tools in, is critical to the equitable monitoring of UN Sustainable Development Goal 2 and to ensure accountability in the guarantee of the rights to an adequate standard of living and health for populations in contexts of extreme vulnerability such as migrants in transit.

## Author contributions

Caroline Deschak (CD): is the first author and with CI participates in the definition of the research question and the methodology of this particular paper. She participated in the definition for the data collection, analysis of data, preparation of the manuscript and discussion of the conclusions and recommendations. CD has given her final approval of the version to be published and have agreed to be accountable for all aspects of the work in ensuring that questions related to the accuracy or integrity of any part of the work are appropriately investigated and resolved.

Cesar Infante (CI): is the corresponding author and with CD developed the objectives, and methodology of the paper; designed data collection strategy; analysis of the data, participated in the preparation of the manuscript, and the definition of the main conclusions and recommendations made for public health policy. CI has given his final approval of the version to be published and has agreed to be accountable for all aspects of the work in ensuring that questions related to the accuracy or integrity of any part of the work are appropriately investigated and resolved.

Verónica Mundo-Rosas (VM): participated in the definition of the analysis of the data, analysis, and preparation of the manuscript. VM has given her final approval of the version to be published and has agreed to be accountable for all aspects of the work in ensuring that questions related to the accuracy or integrity of any part of the work are appropriately investigated and resolved.

Coral A Aragon-Gama (CA): participated in the definition of data collection, data collection, and preparation of the manuscript. CA has given her final approval of the version to be published and has agreed to be accountable for all aspects of the work in ensuring that questions related to the accuracy or integrity of any part of the work are appropriately investigated and resolved.

Manuela Orjuela-Grimm (MO): participated in the definition of the analysis of the data, analysis, and preparation of the manuscript. MO has given his final approval of the version to be published and has agreed to be accountable for all aspects of the work in ensuring that questions related to the accuracy or integrity of any part of the work are appropriately investigated and resolved.

I confirm that this work is original and has not been published elsewhere, nor is it currently under consideration for publication elsewhere. All authors have reviewed the final version of this manuscript and have approved this version for submission. All authors have reviewed the submitted manuscript and approve the manuscript for submission.

## Declaration of Competing Interests

The authors declare that they have no known competing financial interests or personal relationships that could have appeared to influence the work reported in this paper
